# The Importance of Sweet Beverage Definitions When Targeting Health Policies—The Case of Switzerland

**DOI:** 10.3390/nu12071976

**Published:** 2020-07-03

**Authors:** Angelica Sousa, Janice Sych, Sabine Rohrmann, David Faeh

**Affiliations:** 1 School of Agricultural, Forest and Food Sciences HAFL, Bern University of Applied Sciences, Länggasse 85, 3052 Zollikofen, Switzerland; sousa.masf@gmail.com; 2 Institute of Food and Beverage Innovation, ZHAW School of Life Sciences and Facility Management, Einsiedlerstrasse 34, 8820 Wädenswil, Switzerland; janice.sych@zhaw.ch; 3 Division of Chronic Disease Epidemiology, Epidemiology, Biostatistics and Prevention Institute, University of Zurich, Hirschengraben 84, 8001 Zurich, Switzerland; david.faeh@bfh.ch; 4 Health Department–Nutrition and Dietetics, Bern University of Applied Sciences, 8004 Bern, Switzerland

**Keywords:** sugar sweetened beverages, sweet beverages, health policies sociodemographic characteristics, 100% juices, low-calorie sweet beverages, soft drinks, plant-based milk substitutes, Switzerland

## Abstract

Since high-sweet beverage intake is associated with health risks, defining what this term encompasses is relevant to the strategies confronting this problem. This study assessed both the sociodemographic factors associated with sweet beverage consumption in Switzerland and the amount consumed. According to the current definition in Switzerland (SB–CUR), sweet beverages include soft drinks, juices with added-sugar, and low-calorie sweet beverages. Using this definition and the representative menuCH survey (*n* = 2057; ages 18–75), the average daily sweet beverage intake was determined and compared with a new sweet beverage definition (SB–NEW), which included all beverages with free sugars and low-calorie sweeteners. A generalized linear model was used to investigate correlates of sweet beverage consumption. Sweet beverage consumption under the SB–CUR and SB–NEW definition was 240.6 g/day and 329.7 g/day, respectively, with 100% juice consumption accounting for 66% of the difference. Carbonated drinks (sodas), low-calorie sweet beverages, and 100% juices were the highest contributors, each around 60 g/day. SB–NEW intake was higher in individuals who were male, young adults (aged 18–29), from German-speaking regions, obese, or had a lower level of education. As sweet beverage consumption was much higher under the SB–NEW definition, this could have implications for health policies aimed at reducing sugar intake.

## 1. Introduction

Recent WHO guidelines recommend limiting free sugars from all food and beverages to less than 10% of the total energy intake, in order to reduce the associated health hazards [1]. According to the WHO, free sugars encompass all monosaccharides and disaccharides added to foods and drinks by the manufacturer, cook, or consumer, as well as naturally present sugars in honey, syrup, fruit juice concentrates, and 100% fruit juices [1]. However, intrinsic sugars in whole fruits and vegetables, and lactose in milk and milk products are considered to be of lower or no risk and are therefore not included in this definition [2].

Among the wide spectrum of sweet products on the market, sugar-sweetened beverages are major contributors of free sugars to diet. High intake of these beverages has been associated with an increased risk of obesity [3,4], dental caries [5], and major chronic diseases, such as type 2 diabetes [6,7], cardiovascular disease [8,9], and possibly cancer [10].

The WHO defines sugar-sweetened beverages as all beverages containing free sugars, i.e., carbonated or non-carbonated soft drinks, 100% fruit/vegetable juices and drinks, liquid and powder concentrates, flavored water, energy and sports drinks, ready-to-drink tea, ready-to-drink coffee, and flavored milk drinks [11]. Plant-based milk substitutes, which might contain considerable amounts of free sugars, are not included in this definition [12].

In contrast, the majority of studies define sugar-sweetened beverages as beverages with sugars that are added during processing, such as carbonated and non-carbonated soft drinks, fruit drinks, and sports drinks that contain added caloric sweeteners, such as high fructose corn syrup, sucrose, or fruit juice concentrates [4,7,13]. In these studies, however, beverages with naturally present sugars such as 100% juices are excluded.

Another beverage category that is not considered in the above definitions is low or zero calorie sweet beverages. These beverages benefit from increased consumption and might be perceived as healthier choices due to their few or no calories; however, their health effects are controversial [14,15,16]. Their consumption was similarly associated with all-cause mortality, as sugar-sweetened beverages [17] and some low-calorie sweeteners were suggested to increase glucose intolerance by altering the intestinal microbiota [18].

Following the recent WHO recommendation to limit free-sugars intake, many nutritional societies have adapted their sugar recommendations to promote this goal. Additional measures aimed at targeting sweet beverages at the policy level, such as taxes, have also been introduced in a variety of countries [19]. However, in the application of a sugar tax, the WHO definition of sweet beverage does not seem to be consistently applied, leading to the exclusion of several beverages containing free sugars. For example, when defining which beverages should be subject to a sweet beverage tax, the majority of countries apply the tax to beverages with added sugar, but not to beverages containing naturally-present sugars, such as 100% juices (no added sugar) [20,21,22,23,24]. More information on this from a selection of countries can be found in Appendix A: Table A1. Alongside beverages with added sugar, low-calorie sweet beverages are also taxed in some countries, such as France and Philippines [20,22], but not in others [21,23,24] (Appendix A: Table A1).

In Switzerland, the first national representative data on food consumption and dietary behavior, the menuCH survey [25,26,27], was recently conducted and the first report summarized the consumption of all foods and beverages [25]. Average daily intake of sweet beverages was 240.6 g (labeled ‘soft drinks’ in the report) and included the following beverage types—soft drinks, fruit, and vegetable juices with added sugar and low-calorie sweet beverages (corresponding to the current SB–CUR definition used in the present analysis) [25]. However, unlike yoghurts and breakfast cereals for which the food industry has committed to decreasing the quantity of added sugar by 2018 [28], no strategy has been directed at decreasing the quantity of added sugar in sweet beverages in Switzerland.

The above health concerns and the inconsistent application of sweet beverage definitions were the motivation for the present study. Our interest was to explore a new definition of sweet beverages (SB–NEW), which could lead to a more comprehensive assessment of sweet beverage consumption. The SB–NEW definition includes all beverages containing free sugars (as proposed by WHO) with the addition of plant-based milk substitutes and low-calorie sweet beverages. Using the menuCH survey, the aim of the present analysis was to identify the beverages that contribute to sweet beverage consumption, according to two different definitions, i.e., the current definition (SB–CUR) used in Switzerland [25], and SB–NEW as defined above. Through the lens of analysis, the aim was to determine the sociodemographic characteristics associated with this consumption, and to possibly reveal target groups for public health interventions.

## 2. Data and Methods

### 2.1. Data

The data in this analysis were derived from the National Nutrition Survey menuCH, which was conducted in 2014–2015, with a representative sample of 2086 participants aged 18–75-years old from the three main language regions of Switzerland (German, French, and Italian) [25,26,27]. The description and quantification of all foods and beverages consumed, including portion sizes and preparation, were assessed by two 24-hour dietary recalls (24HDR), carried out face-to-face and by telephone. Information about sociodemographic and lifestyle variables was also collected for each study participant. Data of foods, recipes, and ingredients were later linked to the Swiss Food Composition Database, to estimate macronutrient intake [29]. Further information about study design and data collection is provided elsewhere [25,27,30]. Only participants who completed both interviews (2057) were included in this analysis.

Two definitions of sweet beverages were included in this analysis (Table 1)—(1) the current definition used in Switzerland, SB–CUR [25], which included soft drinks, fruit, and vegetable juices, with added sugar and low-calorie sweet beverages; and (2) the new definition proposed in this study, SB–NEW, which included all beverages with free sugars as per the WHO definition, all plant-based milk substitutes as proposed by Swan et al. [12], and low-calorie sweet beverages.

The approach for categorization was adapted from Chatelan et al. [25]. The different sweet beverages identified in menuCH were grouped into five categories (Table 1)—(1) soft drinks included beverages with added sugar; (2) fruit juices and alcohol-free beverages included fruit beverages with added sugars or naturally present sugars; (3) low-calorie sweet beverages included beverages with low- or zero-calorie sweeteners; (4) milk beverages included milk beverages with added sugar and plant-based milk substitutes; and (5) other sweet beverages included remaining beverages such as mineral waters and teas with added sugar. Although coffee drinks with sugar are considered to be sweet beverages by the SB–NEW, these beverages were excluded from the analysis, as it was not possible to identify the beverages that contained added sugar or sweeteners in the dataset.

Milk beverages were only considered sweet beverages if they contained more than 4.7 g sugar per 100 g, which was the quantity of lactose present in 100 g of milk, since lactose is not considered to be a free sugar in the WHO definition [1]. For milk products with added sugar, the amount of lactose (4.7 g/100 g) was removed from the total amount of sugar, to determine the amount of added sugar.

Consistent with previous analyses, which used the menuCH survey [25], the average of the two-day interviews was used to quantify the average daily consumption of sweet beverages (g/day) per person, disaggregated by type of beverage. Mean daily intakes of sweet beverages derived from each definition, SB–NEW and SB–CUR, were compared to identify if the beverages excluded from SB–CUR would be relevant for the overall consumption of sweet beverages.

### 2.2. Data Preparation and Analysis

Dietary data were linked to the sociodemographic characteristics of the study participants. The average daily intake of sweet beverages and its contribution to the total sugar and energy intake were calculated by the selected sociodemographic characteristics. Differences in means between sociodemographic characteristics and between SB–NEW and SB–CUR were tested using ANOVA.

A regression analysis was conducted to identify socio-demographic, anthropometric, and lifestyle characteristics that were associated with the consumption of sweet beverages (g/day). The socio-demographic characteristics considered were sex, age (18–29, 30–44, 45–59, and 60–75 years), language region (German-speaking: Aargau, Basel-Land, Basel-Stadt, Bern, Lucerne, St. Gallen, and Zurich; French-speaking: Geneva, Jura, Neuchatel, and Vaud; and Italian-speaking: Ticino) and level of education (low education—primary and secondary, and high education—tertiary and above). Anthropometric and lifestyle variables included in the analysis were BMI (4 categories—underweight, normal weight, overweight, and obese) and smoking (current smoker and non-smoker). For BMI, international standard protocols were followed for measurements of weight and height [31]. Details of the studied variables were reported earlier [25,30].

Around half of the participants in menuCH did not consume any sweet beverages (SB–CUR). To take this particular aspect of the dependent variable distribution into account, a generalized linear model (GLM) with a log-link function and robust standard errors was used for the analyses [32]. All analyses were weighted for sex, age, marital status, major area of Switzerland, nationality, and household size, to account for the complexity of the survey design [33]. All regressions were also adjusted for the season and the recall day of the week. Regression analyses were conducted for the consumption of SB–NEW, soft drinks, and low-calorie sweet beverages (each at g/day) in the overall population. As a sensitivity analysis, a regression was conducted with the SB–CUR to verify if the population groups at risk of consuming sweet beverages differed between SB–NEW and SB–CUR. All analyses were performed using Stata 11 [34].

## 3. Results

Table 2 shows the sociodemographic characteristics of the study participants—75.7% of adults consumed SB–NEW classified beverages, whereas only 52.5% consumed SB–CUR classified beverages. According to the SB–NEW definition, the average daily consumption of sweet beverages in Switzerland was 329.7 g/day, compared to 240.6 g/day, under the SB–CUR definition (*p* < 0.001) (see Appendix A: Table A2); the majority of the difference (66%) was due to the consumption of 100% juices (no added sugar) (58.6 g/day).

**Figure 1:**
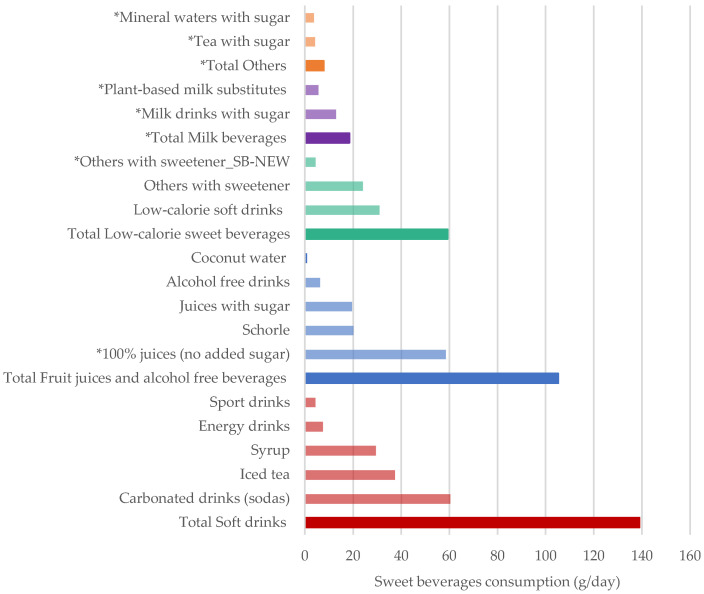
Weighted average consumption of sweet beverages (g/day) based on SB–NEW by sweet beverage category. All results were weighted for sex, age, marital status, major area of Switzerland, nationality, household size, season, and weekday. * Identifies all beverages excluded from the SB–CUR definition such as 100% juices (no added sugar), all milk beverages and all other beverages with added sugar or sweetener. The beverages are listed in ascending order, according to average intake (g/day).

Sweet beverages consumed in the highest amounts were soft drinks (139 g/day), particularly carbonated drinks (sodas) (60.4 g/day), followed by all low-calorie sweet beverages (59.2 g/day), and 100% juices (58.6 g/day) (Figure 1).

**Figure 2:**
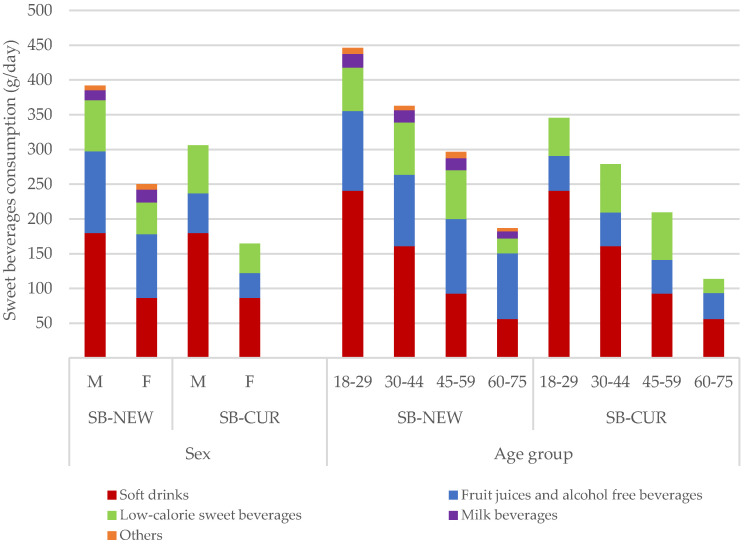
Weighted average consumption of sweet beverages (g/day) SB–NEW vs. SB–CUR by sweet beverage category and by sex and age group. SB–NEW includes all beverages. SB–CUR excludes 100% juices (no added sugar), milk beverages, and other beverages, with added sugar or sweetener, as defined in Table 1. All results were weighted for sex, age, marital status, major area of Switzerland, nationality, and household size. Differences in the means between SB–CUR and SB–NEW by all stratifiers and within stratifiers were statistically significant with ANOVA (*p* < 0.001).

There were statistically significant differences (*p* < 0.001) in the consumption of sweet beverages across sex and age groups, as well as between SB–NEW and SB–CUR (see Figure 2 and Appendix A: Table A2). With both definitions, men consumed 1.6 times more sweet beverages (391.8 g/day SB– NEW and 305.9 g/day SB–CUR) than women (250.3 g/day SB–NEW and 164.7 g/day SB–CUR). In particular, men consumed higher amounts of carbonated drinks (83.3 g/day), iced tea (43.6 g/day), low-calorie soft drinks (40.9 g/day), syrup (38 g/day), “Schorle” (a carbonated mixture of apple juice and water) (28.5 g/day), and sport drinks (11.4 g/day) than women, whereas women consumed twice as much plant-based milk substitute (7.6 g/day) as men.

Adults aged 18–29 years consumed more sweet beverages than any other age group (446.1 g/day and 345.3 g/day, according to SB–NEW and SB–CUR, respectively). This age group consumed particularly high amounts of soft drinks, at around 240.6 g/day. Statistically significant differences were not found for the consumption of 100% juices (no added sugar) across sociodemographic groups, and all groups consumed between 40 and 67 g/day.

**Figure 3:**
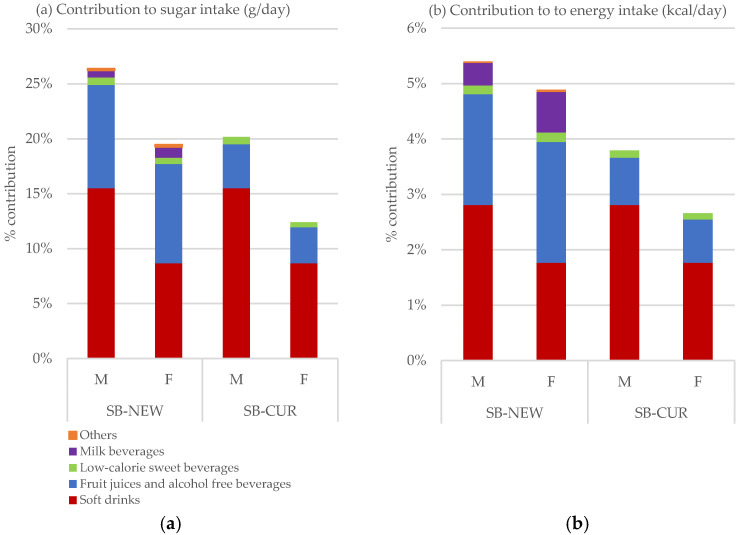
Weighted contribution of sweet beverages estimated by SB–NEW and SB–CUR to total sugar, (g/day) in (**a**) and to energy (kcal/day) in (**b**), from all foods and drinks by sex. SB–NEW includes all beverages; SB–CUR excludes 100% juices (no added sugar), milk beverages and other beverages with added sugar or sweetener, as defined in Table 1. All results were weighted for sex, age, marital status, major area of Switzerland, nationality, and household size. Differences in the means between the SB–CUR and SB–NEW definition by gender were statistically significant with ANOVA (*p* < 0.001).

Considering all foods and drinks, total average consumption of sugar in Switzerland was 106 g/day, and average energy intake was 2225.7 kcal/day (see Figure 3 and Appendix A: Table A3). All differences between men and women, and between SB–NEW and SB–CUR were statistically significant (*p* < 0.001). For men, sweet beverage consumption represented 26.4% (SB-NEW) and 20.2% (SB-CUR) of total sugar consumption, whereas for women, the corresponding contributions to total sugar were 19.5% (SB-NEW) and 12.4% (SB-CUR). The subcategories of 100% juices (5.6%), carbonated drinks (5.4%) and syrup (3.7%) were the highest contributors of total sugar intake. The sugar contribution of these three categories represents 16.7% and 12.3% of the total sugar intake for men and women, respectively (see Appendix A: Table A3).

For men, SB–NEW consumption provided 5.4% of total energy intake (3.8% by SB-CUR), compared with 4.9% (2.7% by SB–CUR) for women.

### Socio-Demographic, Anthropometric, and Lifestyle Correlates with Sweet Beverage Consumption

Based on the regression analysis, several sociodemographic and lifestyle factors were highly associated with the consumption of SB–NEW (see Table 3). In general, men, young adults (18–29-years old), individuals with a lower educational level, individuals living in the German-speaking region and individuals with obesity, consumed significantly more SB–NEW classified beverages. The sensitivity analysis showed that the same sociodemographic and lifestyle factors were significantly associated with the consumption of the beverages defined by SB–CUR.

Men consumed 53.2% more SB–NEW beverages than women. Similarly, adults 18–29-years old consumed more SB–NEW beverages than any other age group (Table 3). Adults aged 30–44, 45–59, and 60–75-years old consumed 18%, 35.3%, and 60.3% less SB–NEW beverages, respectively, than young adults 18–29-years old. Furthermore, people living in the French and Italian regions consumed less SB–NEW beverages than the German-speaking region (25.7% and 38.7%, respectively), and people with a lower educational level consumed 16% more SB–NEW beverages than those with a higher educational level. Finally, individuals with obesity consumed 32.6% more SB–NEW beverages than normal BMI people.

The latter variables were significantly associated with higher consumption of soft drinks and low-calorie sweet beverages (Table 3), except for BMI, which was not significantly associated with the consumption of soft drinks. Similarly, sex and education were not significantly associated with the consumption of low-calorie sweet beverages. In particular, men (103% more than women) and people with a lower educational level (55% more than higher educational level) had a higher consumption of soft drinks. Similarly, smoking (55.5% more than non-smokers) and obesity (148.9% more than normal BMI) were strongly associated with the consumption of low-calorie sweet beverages.

## 4. Discussion

The findings of the present study provide important insights into the consumption levels of sweet beverages, according to two different definitions and reveal sociodemographic and anthropometric characteristics associated with their consumption. It was difficult to compare our data with other studies, due to the varying definitions of sweet beverages. However, the average daily consumption of sweet beverages, estimated by the SB–CUR definition, was 240.6 g/day in Switzerland, compared to 100 g/day in Central Europe (where sweet beverages were defined as carbonated beverages, energy drinks, and fruit drinks) [13]. The higher daily consumption measured with the SB–NEW definition was mainly due to the consumption of 100% juices, which were excluded from the SB–CUR definition. With both definitions (SB–NEW and SB–CUR), the consumption of sweet beverages was higher among men and younger populations, consistent with other studies [35,36].

We found that 100% of the juices were consumed in equally high amounts as carbonated drinks (each around 60 g/day) and both subcategories contributed similarly to the total sugar intake. In the Swiss population, the total sugar from 100% juices and carbonated drinks was twice as high as the corresponding sugar intake in France [37].

Although WHO guidelines specify that 100% juices deliver free sugars, these beverages are still generally perceived as healthier drinks by consumers and represent an important source of vitamins. In some nutritional guidelines, like those provided in Switzerland and the US, one serving of 100% fruit juice could replace one daily portion of fruit or vegetables, based on their vitamin contribution [38,39]. This might partly account for the comparable amounts of 100% juices consumed across sociodemographic groups. Regarding the differences between sugar types, consumers might know that 100% juices do not contain added sugar (sucrose), but are likely less aware that they contain as much sugar (naturally present) and energy as carbonated drinks. There is a great deal of controversy regarding health outcomes related to the consumption of 100% fruit juices [10,14,40,41,42,43]. Their consumption is associated with increased risk of cardiovascular mortality, type 2 diabetes, and cancer [10,14,40]. Beneficial effects from juice intake have been shown in energy-matched substitution studies, while adverse effects have been shown in additional studies, due to the induced excess in energy intake [41]. In contrast, other studies identified positive effects from fruit juice bioactives on oxidative stress and inflammation [42,43].

Given the high consumption of 100% juices and their comparable consumption across sociodemographic subgroups, the Swiss population might benefit from clearer and more consistent information about the quantities of free sugars present in 100% juices, which needs to be considered and counterbalanced with the vitamin contribution of this type of beverages.

### 4.1. Low-Calorie Sweet Beverages and Plant-Based Milk Substitutes

Low-calorie sweet beverages, which represent potential solutions for reducing sugar and energy intake, were consumed at levels as high as 100% juices and carbonated drinks. However, there is considerable controversy concerning the associated health effects of the consumption of these beverages [14,15,16,44]. Emerging evidence shows that non-sugar sweeteners do not contribute to weight loss in adults or children with overweight or obesity actively trying to lose weight [15]. Other outcomes were also linked with consumption of low-calorie sweeteners, such as increased all-cause mortality [17] and increased glucose intolerance [18]. In contrast, evidence for a decreased desire for sweet foods [45], reduced appetite, and energy intake was suggested for some low-calorie sweeteners [46]. Clearly, there is a need to clarify the effects of low-calorie sweeteners, as they continue to be widely used to replace sugars in foods and beverages.

The overall average consumption of plant-based milk substitutes was low but was two-fold higher among women than men. These beverages might be perceived as healthier alternatives than milk, however, they contain considerable amounts of free sugars [12] and have lower nutritional quality compared to cows’ milk [47].

The consumption of both low-calorie sweet beverages and plant-based milk substitutes should be carefully monitored by health authorities. Consumers should also be better informed about their overall composition and possible health risks.

### 4.2. Sociodemographic Characteristics Associated with Sweet Beeverage Consumption

Our analysis showed that the most significant correlates of SB–NEW beverage consumption were male, younger age, lower educational level, living in the German-speaking region, and individuals with obesity. These factors were also associated with the consumption of soft drinks. Similarly, current smokers, individuals with obesity and those living in the German-speaking region had a higher consumption of low-calorie sweet beverages.

The regression analyses for SB–NEW as well as for soft drinks showed higher consumption among men, young adults, and people with a lower educational level in Switzerland; these trends were consistent with previous studies [36,48,49]. Additionally, SB–NEW beverage consumption was higher in the German-speaking region than in the other regions. This supports previous results of dietary pattern analysis, indicating a lower quality diet in the German-speaking region, compared to the French and Italian regions [50].

In contrast to Mullie et al., we found higher SB–NEW beverage consumption among individuals with obesity [51], but did not find a significant association between obesity and soft drink consumption. This might imply that individuals with obesity avoided soft drinks due to their calorie and sugar content, but consumed higher amounts of other sweet beverages in an attempt to drink healthier beverages.

Consistent with Paulsen et al. we found that individuals with obesity consumed more low-calorie sweet beverages than those with a normal weight [49], which is likely linked to the desire to lose weight and avoid sugars and calories. We also found that people currently smoking and living in the German-speaking region consumed more low-calorie sweet beverages.

### 4.3. Strengths and Limitations

The menuCH study is the first national representative data that provides detailed information about the types and amounts of beverage intake in Switzerland. Furthermore, the survey used state-of-the-art dietary assessment tools and included a pilot study to optimize these tools [26]. The interview-led 24 HDR method has many advantages over other self-reported methods, especially relevant for capturing beverage consumption, which is frequently consumed outside of meals and is easily forgotten. Probing by the interviewer helps to retrieve these events [52]. One limitation of the menuCH survey is the absence of a food frequency questionnaire to account for day-to-day variations in food consumption over a longer period. The low participation rate and a high proportion of educated and healthy study participants might have led to an underestimation of our findings, as people with a higher education and better health were overrepresented in the survey [25]. In addition, people were more likely to underreport portion sizes for high-calorie foods, including regular soda [53]. Moreover, underreporting of energy intake was more likely among menuCH participants with a high BMI [54]. It is therefore likely that the consumption of sweet beverages in this study was underreported. Likewise, for dieting reasons, individuals with obesity might replace sweet beverages with low-calorie sweetened beverages, and reverse causation in the association of the latter beverages and obesity could not be excluded [55]. Another limitation was that ready-to-drink coffees containing sugar were excluded from the analyses, as it was not possible to identify the beverages that contained added sugar or sweeteners in the dataset. Since the consumption of these beverages is increasing and new beverages with high amounts of sugar continue to be launched on the market, further analysis is warranted to include ready-to-drink coffees as a new sweet beverage sub-category. In further dietary assessments, additional information, such as brand name and recipe, should be collected to facilitate the quantification of sugars from these beverages. Finally, to be consistent with previous analyses that used menuCH [25], this study used the average of the two-day interviews to quantify the average daily consumption of sweet beverages (g/day) per person. Although methods like MSM or NCI proved to be more robust to estimate usual intake, only minor differences between MSM and the average of the two-day interviews were found [25]. Our results and interpretations would likely not differ based on the method used. In line with the WHO recommendation, this study only focused on sugar and did not take into account other nutritional values. The limitation of this approach is that beverages with different nutritional properties are equally considered based on their free sugar content, regardless of their nutritional value.

Since menuCH was the first survey to collect information on the consumption of sweet beverages, it was not possible to compare trends over time. Thus, further studies should aim to monitor possible changes in sweet beverage consumption patterns of adults in Switzerland over time. Similarly, no data from menuCH were available on the consumption of sweet beverages by children and adolescents, despite new estimates showing that 15% of children in Switzerland were overweight or obese [56]. Thus, further research should be undertaken to determine the rate of consumption of sweet beverages among these population groups.

### 4.4. Policy Implications

The findings of the present study provide insights on the patterns of consumption of sweet beverages, according to two different definitions and across different population groups. Our study showed that the application of the SB–CUR rather than SB–NEW led to a much lower estimation of sweet beverage consumption. This would subsequently impact public health and other policy-measures aimed at reducing sugar intake, which rely on this amount. Effective policies aimed at decreasing sweet beverage consumption included, among others, traffic-light labeling, promotion of healthier beverages in public places, and limiting or removing access to sweet beverages in schools, among other [19]. In particular, sweet beverage taxes were demonstrated to be a cost-effective policy tool for decreasing consumption among populations at risk [11,24,57].

Thus, in addition to following the WHO recommendation to limit free-sugar intake to less than 10% of total energy intake, the SB–NEW definition proposed here might be considered in health-policies aimed at reducing sugar intake, particularly among the target groups identified in this study.

## 5. Conclusions

In Switzerland, the high daily sweet beverage consumption and the substantial variation across different sociodemographic and lifestyle groups call for action to plan and implement strategies towards reducing the consumption of these beverages. While soft drinks and 100% juices were the most consumed beverages contributing to a similar sugar intake, 100% juices are currently not counted as sweet beverages in Switzerland. We also found that low-calorie sweet beverages, considered to be alleged solutions for decreasing sugar intake were consumed in comparable amounts to soft drinks, suggesting that this beverage group might also represent an important public-health target. The proposed SB–NEW definition has the merit of providing a more comprehensive assessment of total sugar intake in liquid form.

This suggests that the application of the new definition, SB–NEW, as well as the close monitoring of sweet beverage consumption will be important measures for developing effective public health policies to reduce sugar intake in Switzerland and in other countries implementing strategies.

## Figures and Tables

**Table 1:** Beverages included in SB–NEW and SB–CUR definition.

Sweet Beverage Category	SB–NEW Definition ^3^	SB–CUR Definition
Soft drinks	Carbonated drinks (sodas)	Carbonated drinks (sodas)
	Iced tea	Iced tea
	Syrup	Syrup
	Energy drinks	Energy drinks
	Sport drinks	Sport drinks
Fruit juices and alcohol-free beverages	100% juices -no added sugar- ^2^ (fruit and vegetable juices and smoothies)	
	“Schorle” (a carbonated mixture of apple juice and water)	“Schorle” (a carbonate mixture of apple juice and water)
	Juices with sugar	Juices with sugar
	Alcohol free drinks	Alcohol free drinks
	Coconut water	Coconut water
Low-calorie sweet beverages ^1^	Low-calorie soft drinks	Low-calorie soft drinks
	Other beverages with sweetener (fruit and vegetable juices, alcohol-free beverages, milk beverages ^2^, tea ^2^ and mineral water ^2^ with sweeteners)	Other beverages with sweetener (fruit and vegetable juices and alcohol-free beverages with sweeteners)
Milk beverages ^2^	Milk drinks with sugar	
	Plant-based milk substitutes ^1^	
Others ^2^	Tea with sugar	
	Mineral waters with sugar	

^1^ Categories excluded from WHO definition. ^2^ Categories excluded from SB–CUR definition. ^3^ Coffee drinks with sugar should be a sub-category of SB–NEW. However, these beverages were excluded from the analysis. SB–NEW: New definition of sweet beverages; and SB–CUR: The current definition used in Switzerland for sweet beverages.

**Table 2:** Sociodemographic characteristics of the study participants, menuCH 2014.

	Total	People Who Consumed, SB–NEW ^1^	People Who Consumed, SB–CUR ^2^
Sex (%)			
Female	50.2	49.3	45.1
Male	49.8	50.7	54.9
Age group (%)			
18–29	18.8	21.3	23.8
30–44	29.9	30.4	33.0
45–59	29.6	28.7	27.6
60–75	21.6	19.6	15.6
Language region (%) ^3^			
German	68.8	70.6	73.4
French	25.7	24.7	22.4
Italian	5.6	4.7	4.2
Education level (%)			
Low education (primary & secondary)	47.4	47.4	47.8
High education (tertiary & above)	52.6	52.6	52.2
Smoking (%)			
Non-smoker	77.6	76.3	74.0
Current smoker	22.4	23.6	26.0
BMI group (%) ^4^			
Normal	54.4	54.8	53.3
Overweight	30.7	30.0	31.4
Obese	12.5	12.6	12.8
Underweight	2.3	2.6	2.5
Sample	2057	1557	1081
Prevalence consumption (%)		75.7	52.5
Weighted sample	4,627,878	3,549,786	2,506,418

^1^ SB–NEW includes all categories of sweet beverages. ^2^ SB–CUR excludes 100% juices (no added sugar), milk beverages, and other beverages with added sugar or sweetener, as defined in Table 1. All results were weighted for sex, age, marital status, major area of Switzerland, nationality, and household size. ^3^ German-speaking region refers to canton Aargau, Basel-Land, Basel-Stadt, Bern, Lucerne, St. Gallen, and Zurich; French-speaking region refers to canton Geneva, Jura, Neuchatel, and Vaud; and Italian-speaking region refers to canton Ticino. ^4^ BMI group: normal (18.5 ≤ BMI < 25 kg/m^2^); overweight (25 ≤ BMI < 30 kg/m^2^); obese (BMI ≥ 30 kg/m^2^); and underweight (BMI < 18.5 kg/m^2^).

**Table 3:** Individual sociodemographic correlates of sweet beverage consumption for SB–NEW, soft drinks, and low-calorie sweet beverages.

Variables	SB–NEW (%) ^3,5^				Soft Drinks (%)				Low-Calorie Sweet Beverages (%) ^4^			
	Coef.	[95% Conf. Interval]			Coef.	[95% Conf. Interval]			Coef.	[95% Conf. Interval]		
Sex (M vs. F)	53.2	[33.5;	75.8]	***	103.0	[59.4;	158.6]	***	36.3	[−4.4;	94.2]	
Age group												
18–29 (ref)	1.0				1.0				1.0			
30–44	−18.0	[−31.3;	−2.3]	*	−29.5	[−46.8;	−6.6]	*	11.3	[−30.4;	77.8]	
45–59	−35.3	[−46.1;	−22.3]	***	−60.9	[−71.1;	−47.1]	***	−7.2	[−42.4;	49.3]	
60–75	−60.3	[−67.2;	−52.1]	***	−77.8	[−84.4;	−68.5]	***	−72.1	[−84.7;	−49.1]	***
Language region ^1^												
German (ref)	1.0				1.0				1.0			
French	−25.7	[−36.6;	−13.1]	***	−25.8	[−44.4;	−1.3]	*	−44.7	[−64.7;	−13.7]	**
Italian	−38.7	[−54.0;	−18.4]	***	−33.4	[−60.1;	10.9]		−43.2	[−72.8;	18.8]	
Low education (primary/secondary vs. tertiary)	16.0	[1.4;	32.6]	*	55.0	[22.8;	95.5]	***	8.9	[−22.2;	52.5]	
Smoking (current smoker vs. non-smoker)	16.2	[−0.3;	35.4]		23.5	[−3.1;	57.5]		55.5	[7.9;	124.1]	*
BMI group ^2^												
Normal (ref)	1.0				1.0				1.0			
Overweight	2.7	[−11.4;	19.1]		11.5	[−12.7;	42.4]		3.0	[−30.6;	53.0]	
Obese	32.6	[5.5;	66.7]	*	27.6	[−17.1;	96.5]		148.9	[56.9;	294.8]	***
Underweight	25.1	[−12.9;	79.8]		21.2	[−22.5;	89.5]		−41.4	[−74.0;	32.1]	
N	2053				2053				2053			

Sources: Authors’ calculation using data from the menuCH. All estimates were produced using a generalized linear model (GLM) with a log-link function and robust standard errors. All models controlled for season and recall day of the week and were weighted for sex, age, marital status, major area of Switzerland, nationality, and household size. Reference indicates the reference group. Statistical significance is indicated with a * *p* < 0.05; ** *p* < 0.01; *** *p* < 0.001. Other covariates such as marital status, nationality, and household status were also explored but not considered for the final analysis, due to multicollinearity, and since they have less explanatory power than the variables finally included in the models. ^1^ German-speaking region refers to canton Aargau, Basel-Land, Basel-Stadt, Bern, Lucerne, St. Gallen, and Zurich; French-speaking region refers to canton Geneva, Jura, Neuchatel, and Vaud; and Italian-speaking region refers to canton Ticino. ^2^ BMI group: normal (18.5 ≤ BMI < 25 kg/m^2^); overweight (25 ≤ BMI < 30 kg/m^2^); obese (BMI ≥ 30 kg/m^2^); and underweight (BMI < 18.5 kg/m^2^). ^3^ SB–NEW definition includes all beverages with free sugars, plant-based milk substitutes, and low-calorie sweet beverages. ^4^ Low-calorie sweet beverages based on SB–NEW definition. ^5^ Reading example: Sex was statistically significant associated with the consumption of SB–NEW; men consumed 53.2% more sweet beverages than women.

## Appendix A

**Table A1:** Example of countries with taxed and untaxed sweet beverages (selection of countries) ^2^.

Country	Taxed Beverages	Untaxed Beverages	Reference
France (2012)	non-alcoholic beverages with added sugar or with sweeteners	100% juices, soda beverages with a min of 2.9% proteins	Service-Public France 2019
United Kingdom (2018) “Soft Drinks Industry Levy”	beverages with added sugar ^1^ during production; contains at least 5 g of sugar per 100 mL in its ready to drink or diluted form, has a content of 1.2% alcohol by volume or less	100% juices, milk replacements (e.g., almond), zero-calorie beverages with sweeteners such as aspartame and stevia	UK-Gov 2019
Philippines (2017)	beverages made with caloric and non-caloric sweeteners	100% natural juices and 3 in 1 instant coffee	Saxena A. 2019
Chile (2014)	beverages that contain at least 6,25 g of added sugar per 100 mL	reduced the tax of beverages with low added sugar less than 6.5 g per 100 mL such as soda drinks with sweeteners	Cuadrado C. 2018
Mexico (2013)	non-dairy and non-alcoholic beverages with added sugar beverages (sodas, flavored and sweetened juices) or with more than 275 kcal per 100 g	diet sodas and sparkling water, flavored water without caloric sugar, 100% fruit juices and beer	Colchero MA et al. 2016

^1^ added sugar defined as mono or disaccharides, or anything (other than fruit juice, vegetable juice and milk) that contains sugar, such as honey. ^2^ These countries were chosen because they exemplify the different definitions on sugar sweetened beverages used for policy making.

**Table A2:** Weighted-average consumption of sweet beverages (g/day) by gender and age group.

Sweet Beverage Categories	Total Amount (g/day)	Sex				Age Group							
		F		M		18–29		30–44		45–59		60–75	
	Mean	Mean	%	Mean	%	Mean	%	Mean	%	Mean	%	Mean	%
**Soft drinks**	139.0	86.5	35	179.8	46	240.6	54	160.9	44	92.7	31	56.2	30
Carbonated drinks (sodas)	60.4	33.7	13	83.3	21	92.5	21	64.6	18	52.7	18	27.8	15
Iced tea	37.3	24.5	10	43.6	11	74.4	17	42.0	12	18.7	6	8.8	5
Syrup	29.4	22.7	9	38.0	10	48.7	11	38.4	11	18.4	6	19.6	10
Energy drinks	4.3	3.1	1	3.5	1	7.6	2	5.8	2	0.5	0	0.0	0
Sport drinks	7.5	2.6	1	11.4	3	17.3	4	10.1	3	2.4	1	0.0	0
**Fruit juices and alcohol-free beverages**	105.2	91.5	37	117.8	30	114.9	26	102.7	28	107.4	36	94.6	51
100% juices (no added sugar) ^2^	58.6	55.8	22	60.5	15	64.6	14	54.0	15	58.7	20	57.4	31
Schorle	20.2	13.0	5	28.5	7	12.4	3	22.5	6	26.1	9	17.8	10
Juices with sugar	19.4	18.3	7	20.5	5	34.9	8	21.7	6	12.9	4	11.7	6
Alcohol free drinks	6.1	3.7	1	7.2	2	2.6	1	1.7	0	9.6	3	7.4	4
Coconut water	0.9	0.7	0	1.1	0	0.3	0	2.7	1	0.0	0	0.3	0
**Low-calorie sweet beverages**	59.2	45.9	18	73.4	19	62.5	14	75.3	21	70.1	24	21.1	11
Low-calorie soft drinks	30.9	22.2	9	40.9	10	37.6	8	37.0	10	37.1	13	10.8	6
Others with sweetener	24.1	20.2	8	27.9	7	16.9	4	32.3	9	30.9	10	9.4	5
Others with sweetener-SB-NEW ^2^	4.3	3.5	1	4.6	1	8.0	2	5.9	2	2.0	1	0.9	0
**Milk beverages ^2^**	18.5	18.4	7	14.3	4	19.7	4	17.7	5	17.3	6	10.3	5
Milk drinks with sugar	12.8	10.8	4	10.9	3	15.3	3	12.3	3	9.1	3	7.7	4
Plant-based Milk substitutes	5.6	7.6	3	3.3	1	4.4	1	5.5	2	8.2	3	2.6	1
**Others ^2^**	7.8	7.9	3	6.5	2	8.5	2	6.2	2	9.1	3	4.8	3
Tea with sugar	4.1	4.2	2	3.5	1	5.3	1	3.9	1	4.6	2	1.5	1
Mineral waters with sugar	3.7	3.7	1	3.0	1	3.2	1	2.3	1	4.5	2	3.3	2
SB-CUR ^2^	240.6	164.7		305.9		345.3		278.9		209.4		113.7	
SB-NEW ^1^	329.7	250.3		391.8		446.1		362.8		296.5		187.0	

^1^ SB–NEW includes all sweet beverages. ^2^ SB–CUR excludes 100% juices (no added sugar), milk beverages, and other beverages with added sugar or sweetener, as defined in Table 1. All results were weighted for sex, age, marital status, major area of Switzerland, nationality, and household size. The columns with % represent the contribution of the specific beverage consumption (g/day) to the total SSB–NEW consumption (g/day). The data were listed in ascending order, according to average intake (g/day). Differences in the mean values between SB–CUR and SB–NEW in all stratifies, were statistically significant as per ANOVA (*p* < 0.001).

**Table A3:** Weighted contribution of sweet beverages to total sugar (g/day) and energy intake (kcal/day) from all foods and drinks, by gender.

Sweet Beverage Categories	Contribution to Sugar Intake (g/day)						Contribution to Energy Intake (kcal/day)						Sample
	Female		Male		Overall		Female		Male		Overall		
	Mean	(%)	Mean	(%)	Mean	(%)	Mean	(%)	Mean	(%)	Mean	(%)	
**All foods and drinks -mean (SD)-**	95.8 (44.3)		113.3 (60.7)		106 (54.3)		1904.7 (538.3)		2518.2 (788)		2225.7 (752.8)		2057
**Soft drinks**	8.3	8.7	17.6	15.5	13.4	12.7	33.6	1.8	70.8	2.8	54.0	2.4	682
Carbonated drinks (sodas)	3.2	3.3	7.9	6.9	5.7	5.4	12.8	0.7	31.6	1.3	23.1	1.0	427
Iced tea	1.8	1.9	3.1	2.8	2.7	2.6	7.2	0.4	12.7	0.5	10.9	0.5	205
Syrup	3.0	3.2	5.0	4.4	3.9	3.7	12.3	0.6	20.2	0.8	15.7	0.7	176
Energy drinks	0.3	0.3	1.3	1.1	0.8	0.8	1.2	0.1	5.1	0.2	3.4	0.2	42
Sport drinks	0.0	0.0	0.3	0.3	0.3	0.2	0.2	0.0	1.2	0.0	1.1	0.0	14
**Fruit juices and alcohol-free beverages**	8.6	9.0	10.6	9.4	9.7	9.2	41.6	2.2	50.4	2.0	46.3	2.1	1094
100% juices (no added sugar) ^2^	5.5	5.8	6.1	5.4	5.9	5.6	26.7	1.4	28.9	1.1	28.1	1.3	874
Schorle	0.9	1.0	2.0	1.8	1.4	1.4	4.0	0.2	8.8	0.4	6.3	0.3	117
Juices with sugar	1.9	2.0	2.3	2.0	2.1	2.0	9.4	0.5	10.6	0.4	9.9	0.4	264
Alcohol free drinks	0.2	0.3	0.2	0.2	0.3	0.3	1.3	0.1	1.7	0.1	1.8	0.1	88
Coconut water	0.0	0.0	0.1	0.1	0.1	0.1	0.2	0.0	0.3	0.0	0.3	0.0	5
**Low-calorie sweet beverages**	0.6	0.6	0.8	0.7	0.7	0.7	3.3	0.2	4.0	0.2	3.9	0.2	305
Low-calorie soft drinks	0.0	0.0	0.0	0.0	0.0	0.0	0.0	0.0	0.0	0.0	0.0	0.0	165
Others with sweetener	0.4	0.5	0.7	0.6	0.6	0.6	2.1	0.1	3.3	0.1	2.8	0.1	139
Others with sweetener SB-NEW ^2^	0.1	0.1	0.1	0.0	0.1	0.1	1.1	0.1	0.7	0.0	1.0	0.0	41
**Milk beverages ^2^**	0.9	1.0	0.7	0.6	0.9	0.9	13.9	0.7	10.2	0.4	13.6	0.6	260
Milk drinks with sugar	0.5	0.5	0.6	0.5	0.6	0.6	8.0	0.4	7.8	0.3	9.3	0.4	153
Plant-based Milk substitutes	0.4	0.4	0.2	0.1	0.3	0.3	5.9	0.3	2.3	0.1	4.2	0.2	112
**Others ^2^**	0.2	0.2	0.2	0.2	0.2	0.2	0.8	0.0	0.7	0.0	0.8	0.0	77
Tea with sugar	0.1	0.1	0.1	0.1	0.1	0.1	0.5	0.0	0.5	0.0	0.5	0.0	48
Mineral waters with sugar	0.1	0.1	0.1	0.0	0.1	0.1	0.3	0.0	0.2	0.0	0.3	0.0	29
**SB-CUR ^2^**	11.9	12.4	22.8	20.2	17.9	16.9	50.6	2.7	95.6	3.8	75.1	3.4	1081
**SB-NEW ^1^**	18.6	19.5	29.9	26.4	25.0	23.7	93.2	4.9	136.0	5.4	118.6	5.3	1557

^1^ SB–NEW includes all sweet beverages. ^2^ SB–CUR excludes 100% juices (no added sugar), milk beverages, and other beverages with added sugar or sweetener, as defined in Table 1. SD in parenthesis. All results were weighted for sex, age, marital status, major area of Switzerland, nationality, and household size. German-speaking region refers to canton Aargau, Basel-Land, Basel-Stadt, Bern, Lucerne, St. Gallen, and Zurich; French-speaking region refers to canton Geneva, Jura, Neuchatel, and Vaud; and Italian-speaking region refers to canton Ticino. The columns with % report the contribution of each beverage to the total sugar and energy intake of all foods and drinks—mean—(first line of the table). The data were listed in ascending order, according to average intake (g/day). Differences in the mean values between the SB–CUR and SB–NEW definition, in all stratifies, were statistically significant, as per ANOVA (*p* < 0.001).
